# Book Review

**DOI:** 10.1120/jacmp.v7i2.2253

**Published:** 2006-05-25

**Authors:** Srinivasan Vedantham

**Affiliations:** ^1^ Assistant Professor of Radiology, and Andrew Karellas Ph.D., Professor of Radiology The Winship Cancer Institute at Emory University 1701 Upper Gate Drive NE, Suite C5018 Atlanta Georgia 30322 U.S.A.

## Abstract

*Practical Radiography*, by Peter H. Hertrich, Publicis Corporate Publishing, John Wiley Publishers Inc. (U.S.A.), 2005, ISBN 3‐89578‐210‐6, list price $62.23 (web)

This current edition of *Practical Radiography: Principles and Applications* by Peter H. Hertrich traces its roots back to the training manual by Erwin A. Hoxter in 1943 and subsequently Hoxter's *Practical Radiography*, the 6th edition of which was published in 1953. Subsequent editions of *Practical Radiography* expanded to include new technological information; the previous edition was published in 1991. Considering that approximately 15 years have passed since the previous edition and the growth in X‐ray imaging technology, notably in modern digital detectors, this edition is timely. This current edition, as with its predecessors, is primarily intended as an introductory text for X‐ray service personnel, equipment developers, equipment sales personnel and technologists, and, to some extent, radiologists to provide a perspective on the technical aspects of X‐ray imaging. This book could also serve both as a primer for undergraduate level students seeking a career in medical X‐ray imaging and an introductory book for people transitioning to medical X‐ray imaging from other fields. However, this alone may be insufficient because discussions on the theoretical aspects of imaging physics and image science including quality metrics are at a minimum. It would be beneficial for readers with specific interest in these matters to refer to more appropriate literature.

This book provides a practical and technical viewpoint of X‐ray imaging and is well organized into 10 chapters:

1. Medicine and Technology
2. The Physical Principles
3. The Characteristic of X‐rays
4. The Quality of X‐rays
5. Dose
6. X‐ray Systems for Diagnostics and Intervention
7. X‐ray System Components
8. Image Receptor Systems
9. X‐ray Imaging Technology
10. Patient Data Management

Each chapter provides a concise presentation of the material and is adequately supplemented with illustrations, pictures, and images, all of which are good quality with the exception of a few early pictures, which is understandable. The book is almost devoid of mathematics; this may be acceptable considering that the focus is to provide an overview of the technical aspects of X‐ray imaging to a diverse readership. Further, the definition of detective quantum efficiency on pages 147 and 178 is cursory, and the readers would benefit from a more rigorous text on such metrics.

In the approximately 15 years between the current and previous editions, the most noticeable improvement in X‐ray imaging has been in the field of electronic imaging receptors, commonly referred to as digital detectors. The book addresses the basics of image formation in such receptors for most of the major technologies that are currently in use but does not address important factors such as pixel matrix sampling and any associated aliasing. Overall, in our opinion, this book provides a good overview of technical aspects of X‐ray imaging and is best suited for the primary intended audience: service personnel, equipment developers, and sales personnel. In our opinion, this book could also be of interest to technologists, radiologists, freshman level college students, and people transitioning to medical X‐ray imaging from other fields, with substantial supplementation from texts more relevant to their expertise and career path.

